# C-peptide: an essential ally in microvascular complications of type 2 diabetes mellitus and obesity

**DOI:** 10.1186/s13098-024-01454-1

**Published:** 2024-08-29

**Authors:** Regina Esze, Sándor Barna, Péter Fülöp, Péter Kempler, Márton Mikó, Dénes Páll, György Paragh, Sándor Somodi, Miklós Emri, Zita Képes, Ildikó Garai, Miklós Káplár

**Affiliations:** 1 Division of Metabolic Diseases, Department of Internal Medicine, Faculty of Medicine, University of Debrecen, Nagyerdei Krt. 98, Debrecen, 4032 Hungary; 2 Kálmán Laki Doctoral School, Faculty of Medicine, University of Debrecen, Nagyerdei Krt. 98, Debrecen, 4032 Hungary; 3 Division of Nuclear Medicine and Translational Imaging, Department of Medical Imaging, Faculty of Medicine, University of Debrecen, Nagyerdei Krt. 98, Debrecen, 4032 Hungary; 4 Department of Medicine and Oncology, Semmelweis University, Korányi Sándor U. 2/a, Budapest, 1083 Hungary; 5 Department of Medical Clinical Pharmacology, Faculty of Medicine, University of Debrecen, Nagyerdei Krt. 98, Debrecen, 4032 Hungary

**Keywords:** C-peptide, Microcirculation, Neuropathy, Single-photon emission computed tomography (SPECT), Type 2 diabetes mellitus, Obesity

## Abstract

**Background:**

In order to investigate microvascular complications in metabolic diseases, we aimed to investigate cerebral and peripheral microcirculation in relation to peripheral neuropathy and laboratory biomarkers in type 2 diabetes mellitus (T2DM) and obesity.

**Methods:**

Based on the degree of neuropathy (NP), study participants (40 T2DM and 30 obese individuals) were classified into no-NP, mild-NP and severe-NP subgroups. After the injection of Technetium-99 m hexamethylpropylene amine oxime, both T2DM and obese participants underwent single-photon emission computed tomography/computed tomography ([99mTc]Tc-HMPAO SPECT/CT) and SPECT-only examinations to assess lower limb and brain perfusion; respectively. Peripheral nerve function was evaluated with a neurometer and glycaemic markers were measured from plasma in both groups.

**Results:**

Compared to the obese individuals, lower extremity perfusion was significantly reduced in the diabetic subjects *(p* < *0.005*), while it showed a positive correlation with C-peptide levels and negative association with HbA1c values. A U-shape pattern of peripheral microcirculation was observed between the NP groups, indicating a surprisingly better perfusion in the severe-NP group than in the mild one, with the highest levels in obese patients. Since changes in the C-peptide levels exhibited a similar U-shaped trend across the NP subgroups, we suggest a positive correlation between C-peptide levels and the extent of peripheral perfusion. Although, C-peptide values and cerebral microcirculation correlated positively (*rho* = *0.27)*, brain perfusion did not show any differences neither between the diabetic and the obese patients, nor between the NP subgroups *(at p* < *0.05).*

**Conclusions:**

Establishing the link between neuropathy and peripheral microcirculation, C-peptide seems to be a promising biomarker for the prediction of microvascular alterations in metabolic diseases. Of note, the dominance of metabolic factors over microvascular damage in the development of obesity-related neuropathy should be emphasized as well.

## Introduction

Accumulating evidence reports that altered microcirculation in type 2 diabetes mellitus (T2DM) and obesity impose a significant burden on healthcare systems, which is deemed to worsen in the upcoming years [1–3]. Cerebral microvascular impairments induced by metabolic dysfunction lead to perfusion abnormalities that represent an increased likelihood for the development of cognitive dysfunction and neurodegenerative disorders [4–8]. Dementia-related deterioration of memory, poorer executive functioning or diminished daily functional activities result in a substantial drop of life quality.

and reduced life expectancy [9–12]. Beyond central involvement, peripheral vasculopathy manifested in form of neuropathy (NP) is another life-threatening microvascular alteration in association with metabolic diseases. Peripheral neuropathy (PN) not only affects more than the half of the diabetics, but also shows a firm relationship with obesity [13–16]. The devastating disease symptoms, such as lower limb pain, sensory deficits, or gait instability make PN one of the key contributors to reduced level of well-being and disability [17, 18]. In addition, foot ulcers developed on the basis of PN could be responsible for the majority of lower extremity amputations in metabolic diseases [19, 20]. Considering the above mentioned, timely diagnosis of disease-related microvascular changes seems pivotal [1, 2, 21, 22]. Nuclear medical techniques, including single-photon emission computed tomography (SPECT) offer unique opportunity to evaluate central and peripheral microcirculation in a non-invasive and quantitative manner [23, 24]. Based on previous findings, Technetium-99 m hexamethylpropylene amine oxime ([^99m^Tc]Tc-HMPAO) brain SPECT imaging appears to be a useful tool in the detection of subtle brain perfusion abnormalities in patients with type 2 diabetes mellitus or obesity [25, 26]. Furthermore, [^99m^Tc]Tc-HMPAO SPECT/CT (single-photon emission computed tomography/computed tomography) imaging was found to be feasible to imply microvascular alterations triggered by metabolic diseases [27].

To assess peripheral nerve dysfunction, associated with T2DM and obesity, neurometer-based measurement of current perception threshold (CPT) has been proposed. This semi-objective technique allows the non-invasive evaluation of the conduction and the functional integrity of unmyelinated, small myelinated and myelinated nerve fibers [28–31].

Aside from the routinely used glycaemic parameters, including plasma glucose, insulin and glycated haemoglobin (HbA1c), connecting peptide (C-peptide) has recently gained increasing attention in metabolic diseases. As a cleavage product of proinsulin, the 31 amino acid-based C-peptide is released from the pancreas during insulin secretion [32]. Given its inherent ability to correctly represent pancreatic beta cell function [33], C-peptide seems to be a suitable biomarker of insulin secretion, making its measurement a useful tool for therapeutic decision-making in contemporary clinical practice [33, 34]. Also, recent findings indicate that C-peptide measurement has additional beneficial effects in the prevention and improvement of diabetic vasculopathy as well as visceral complications [35]. C-peptide also enhances nitric oxide production that leads to the reduction of vascular resistance and vasodilation [36]. Although assessment of C-peptide levels has demonstrated considerable promise in the management of type 1 diabetes mellitus and advanced stages of T2DM, its influence in earlier disease periods has not yet been settled [35, 37]. Additionally, obesity is usually accompanied by hyperinsulinemia and increased C-peptide levels.

Research on disease pathophysiology using such diagnostic tools would not only contribute to the identification of novel biomarkers and potential therapeutic targets but may eventually result in the establishment of personalized patient management and the implementation of effective prevention strategies. Prior studies have linked both diabetes and obesity with microvascular impairments and peripheral neuropathy [14–16]. In addition, several researchers have published the beneficial effects of C-peptide on microcirculation and observed significant changes in its serum concentrations in these metabolic diseases, establishing the basis for the comparison of such high-risk patient populations. Thus, we aimed to investigate central and peripheral microcirculation and sensory nerve function of type 2 diabetic and obese patients. We tried to explore whether the severity of peripheral neuropathy could be associated with the development of both peripheral and central microvascular impairments in patients with these metabolic disorders, and tried to assess to what extent anthropometric and glycaemic parameters influence these possible associations.

## Materials and methods

### Study design and population

Forty patients with T2DM and 32 obese individuals were assigned in this multimodal clinical study. Eligible participants—aged between 18 and 70 years – were divided into two groups: Regularly controlled obese (without diabetes, Body Mass Index (BMI) > 30 kg/m2) or type 2 diabetic patients (regardless of body weight), from the Division of Metabolic Diseases, Department of Internal Medicine, Faculty of Medicine, University of Debrecen (Debrecen, Hungary), and from a private general medical practice of Borsod-Abaúj-Zemplén county (Miskolc, Hungary). Candidates with anamnestic data on mental or brain disorders as well as peripheral arterial disease (PAD) *(confirmed by physical examination or handheld Doppler ultrasonography)* were excluded. Other key exclusion criteria were as follows: pregnancy, breastfeeding, acute or chronic inflammatory diseases, liver diseases, diagnosed hyperthyroidism or uncontrolled hypothyroidism, history of malignant diseases (except for basocellular carcinoma), presence of crural ulcer, ongoing oral steroid or retinoid treatment, long-term anticoagulant therapy, or any change in regular medical treatment within six months before the commencement of the study. After providing detailed information about the research objectives and the examinations, all participants provided written informed consent. The study was approved by the relevant ethics committee (OGYEI/2829–4/2017).

### Assessment of anthropometric parameters

Anthropometric assessment included body weight (BW), height, and BMI measurement. Standing height was determined with outpatient medical scales to the nearest 0.1 cm (cm). BW was measured using a standard digital scale (MMSZ1, Micra Metriopond Ltd., Hódmezővásárhely) with a maximum weight capacity of 150 kg (kg) and accurate to 0.1 kg. Participants were weighed barefoot in light clothing. Height and weight measurements were presented in cm and kgs, respectively, to one decimal place. BMI was calculated as the BW in kgs divided by height in square meters (m^2^). The age of all participants was documented in years.

### Measurement of laboratory parameters

To assess the actual glycaemic status of the study participants, baseline plasma glucose, HbA1c, C-peptide and insulin levels were determined. Blood samples were collected in fasting state and immediately processed in the Institute of Laboratory Medicine, Faculty of Medicine, University of Debrecen (Debrecen, Hungary) under standardized conditions. Plasma glucose concentrations (reference range: 3.6–6.0 mmol/L) were determined from plasma samples containing sodium fluoride-potassium oxalate (NaF–KOx) The measurement of HbA1c levels was achieved through high-performance liquid chromatography (HPLC) method (BioRad, Hercules, CA, USA) using K3-EDTA anticoagulated whole blood samples. The reference range for HbA1c was set at 4.2–6.1%. Native plasma samples were applied for the quantification of C-peptide (reference: 350–1170 pmol/L) and insulin (reference: 4.3–20 mU/L) levels.

### Single-photon emission computed tomography (SPECT) perfusion studies

#### Assessment of peripheral perfusion

**Fig. 1:**
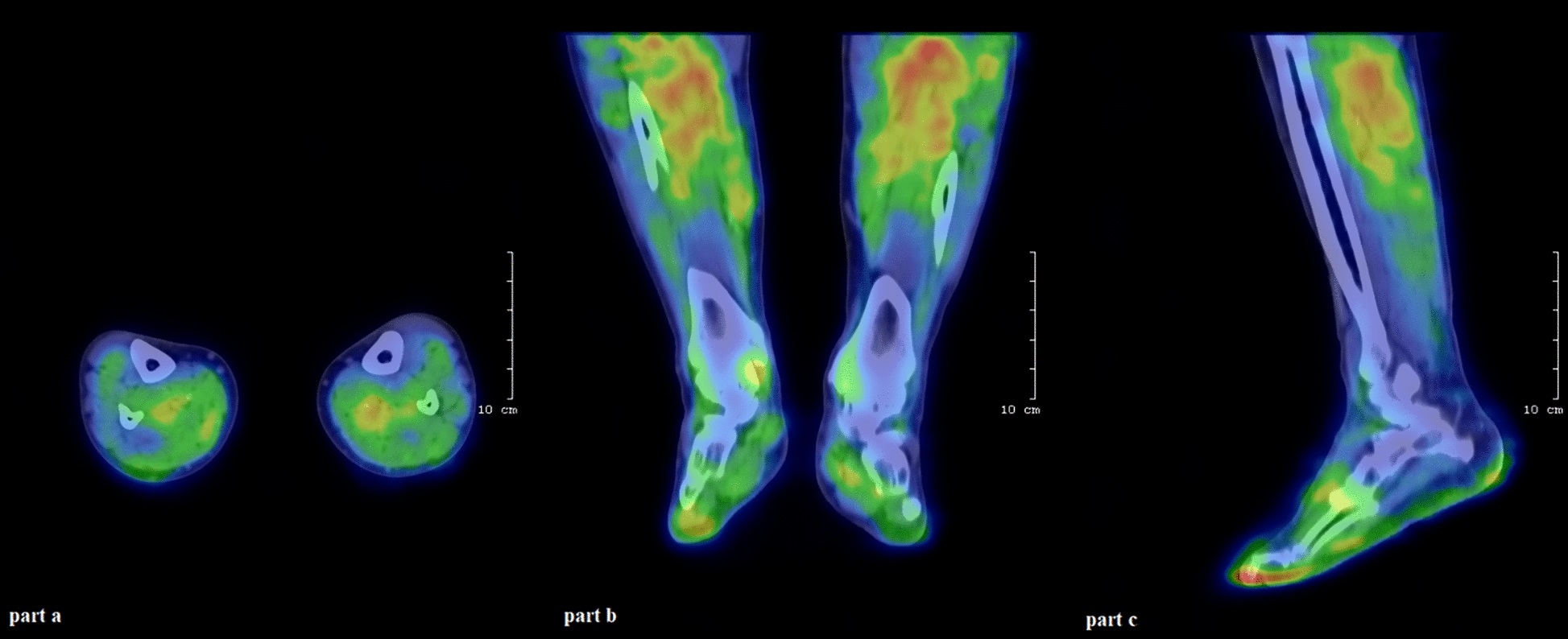
Fused transaxial (**a**), coronal (**b**), sagittal (**c**) [99mTc]Tc-HMPAO SPECT/CT images of the lower extremities obtained 15 min post tracer injection with AnyScan^®^ TRIO SPECT/CT device (Mediso Ltd., Budapest, Hungary). The colours indicate the degree of the radiopharmaceutical uptake of the lower limbs that well correlates with perfusion. (The brighter the color (red) the better perfused the region is). *HMPAO* hexamethylpropylene amine oxime, *SPECT/CT* single-photon emission computed tomography/computed tomography

The examination of lower limb perfusion using SPECT/CT occurred based on the protocol described earlier by Képes et al*.* [27]. Briefly, 15 min after the intravenous administration of approximately 709 ± 42 MBq [^99m^Tc]Tc-HMPAO, Mediradiopharma, Budapest, Hungary) into the right cubital vein of the patients, lower limb SPECT/CT imaging was accomplished in supine position using AnyScan® TRIO SPECT/CT device (Mediso Ltd., Budapest, Hungary). The frame covered a 40 cm long area proximal to the feet of both lower extremities encompassing the calf muscles. Thirty minutes prior to the acquisition 1000 mg perchlorate capsule was given per os to all study participants to prevent the unintended radiotracer uptake of the thyroid glands. For attenuation correction low-dose CT (computed tomography) was used, and image post-processing was done with Tera-Tomo Q SPECT reconstruction method (Mediso Ltd., Budapest, Hungary). To quantify the tracer uptake of the lower limbs, volumes of interest (VOIs) with a diameter of 1 cm were manually placed over the sural muscles (as seen in Fig. 1). Thereafter, mean standardized uptake values (SUV_mean_) were registered in each VOI using Interview Fusion™ image analyses software (Mediso Ltd., Budapest, Hungary).

Pots [^99m^Tc]Tc-HMPAO imaging of the lower limbs, standard brain perfusion SPECT scan was carried out for the assessment of central microcirculation.

#### Assessment of central perfusion

After lower limb SPECT/CT examination, brain SPECT imaging was performed on all study participants using AnyScan® S SPECT camera (Mediso Ltd., Budapest, Hungary) without the injection of further radiopharmaceutical. To achieve baseline state of relaxation participants spent a 10-min rest in a dimly-lit room before imaging. Overall, four patients did not give their consent to undergo brain perfusion SPECT studies.

### Nerve conducting studies

For the quantitative evaluation of sensory nerve fiber functionality, CPT values of the big toes and thumbs were determined using a neurometer (NM-01/CPT; MSB-MET Ltd., Walldorf, Germany). Transcutaneous electric stimulations were manually applied three times at frequencies of 2000 Hz, 250 Hz, and 5 Hz to both big toes (L.V. fiber) and thumbs (C.VI. fiber) to trigger the peroneal and the median nerves, respectively. Conductive gel-coated electrodes attached to the skin served as medium for electrical transmission. To automatically generate CPT values at each examined frequencies, participants were instructed to press a button upon feeling the current stimulus. Mean CPT values were 330 ± 79.5 at 2000 Hz, 123 ± 38.8 at 250 Hz, and 76 ± 31.9 at 5 Hz on the peroneal and 230 ± 55.1, 88.5 ± 27.8, and 47.5 ± 18.8 at 2000, 250 and 5 Hz; respectively on the median nerve. The severity of NP depended upon how far the CPT value—expressed in standard deviation (SD)—was from the median.

Patients were classified into three distinct groups based on the SD deviation of the CPT value from the mean value. Patients with CPT values within the range of the mean ± 2 SD were considered non-neuropathic (no neuropathy subgroup, no-NP). Patients whose CPT values were between the mean CPT + 2 SD and the mean CPT + 4 SD belonged to the mild neuropathy (mild-NP) subclass. Patients were considered to have severe neuropathy (severe-NP) if their CPT values exceeded the mean by more than 4 SD or if they exhibited anaesthesia (extremely high CPT). The CPT-based patients’ classification as outlined in Table 1.

**Table 1:** Baseline characteristics of the NP subgroups

Parameters	No neuropathy		Mild neuropathy		Severe neuropathy	
	Type 2 diabetic participants	Non-DM, obese participants	Type 2 diabetic participants	Non-DM, obese participants	Type 2 diabetic participants	Non-DM, obese participants
Number of patients	5	6	7	3	28	23
Age (years)	49.8 ± 8.26	56.17 ± 8.89	48.43 ± 7.00	51.0 ± 16.09	51.82 ± 6.79	50.3 ± 10.24
BMI (kg/m^2^)	36.32 ± 8.71	39.82 ± 7.53**	30.61 ± 3.33	35.26 ± 1.69**	32.94 ± 4.51	39.01 ± 6.08**
Glucose (mmol/L)	10.0 ± 1.77	5.52 ± 0.8**	8.24 ± 3.33	5.13 ± 0.29**	8.97 ± 3.68	5.46 ± 0.46**
HbA1c (%)	8.3 ± 1.03	5.65 ± 0.23**	7.31 ± 1.13	5.17 ± 0.15**	7.56 ± 1.2	5.55 ± 0.29**
C-peptid (pmol/L)	724.2 ± 380.02	717.83 ± 357.83*	705.43 ± 634.14	415.00 ± 122.88*	753.18 ± 447.94	948.39 ± 413.11*
Insulin (mU/L)	13.5 ± 12.44	17.17 ± 7.28*	13.98 ± 14.12	6.23 ± 3.69*	13.54 ± 9.64	19.2 ± 10.8*

Data are presented as mean ± SD. Asterisk indicates significance between the investigated variables that were significantly different between the Type 2 diabetic and the Non-DM obese groups within the different NP subclasses: *p* ≤ 0.05 (*) and p ≤ 0.01 (**)

*BMI* body mass index, *C-peptide* connecting peptide, *DM* diabetes mellitus *HbA1c* glycated haemoglobin, *NP* neuroparthy, *SD* standard deviation

### Statistical analyses

Given that numerous parameters displayed a distribution that deviated from the Gaussian, we opted for non-parametric techniques to perform statistical analyses. Specifically, we utilized Wilcoxon rank sum test to evaluate group differences and Spearman correlation tests were conducted for the examination of potential monotonic relationships between parameter pairs. R software (version 4.3.2) was used for all statistical analyses, as well as for the generation of tables and figures.

## Results

### Patient characterization—baseline assessment

The ages of the sample population ranged from 32 to 73 years. The mean age was found to be 50.98 ± 6.95 years for the diabetics and 51.47 ± 10.45 years for the obese participants. The total study population composed of 72 subjects including 40 patients with T2DM and 32 obese individuals. There were 24 and 14 men in the T2DM and obese groups; respectively, while 16 diabetic and 18 obese women were enrolled in the study.

First, all/both measured anthropometric (age, BMI) and laboratory data were compared between the two study groups, thereafter correlation analyses were accomplished between the baseline parameters and the results of the perfusion studies as well as the nerve conducting tests. No significant difference was revealed between either the age *(p* = *0.57)* or the sex *(p* = *0.26)* of the type 2 diabetic and the obese participants. As displayed by Table 2, BMI differed significantly between the obese and the diabetic patients being higher in the obese group (38.81 ± 6.08) in comparison with the T2DM cohort (32.95 ± 5.10; *p* < *0.001)*.

**Table 2:** Anthropometric data and glycaemic biomarkers of patients with obesity and type 2 diabetes mellitus

Parameters (reference ranges)	Type 2 diabetic participants (n = 40)		Non-DM, obese participants (n = 32)	
	Mean	SD	mean	SD
Age (years)	50.98	6.95	51.47	10.45
BMI (18,5–24,9 kg/m^2^)	32.95	5.10	38.81**	6.08
HbA1c (4.2–6.1%)	7.61	1.18	5.53**	0.29
Glucose (3.6–6 mmol/L)	8.97	3.41	5.44**	0.52
C-peptide (350–1170 pmol/L)	741.20	464.71	855.16*	414.23
Insulin (4.3–20 mU/L)	13.61	10.53	17.61*	10.33

Asterisks indicate those parameters that were significantly different between the two groups*: p* ≤ 0.05 (*) and p ≤ 0.01 (**)

*BMI* body mass index, *C-peptide* connecting peptide, *DM* diabetes mellitus, *HbA1c* glycated haemoglobin, *SD* standard deviation

Analysing the glucose homeostasis of our sample, higher fasting blood glucose levels were found in the T2DM group as compared to the obese with respective values being 8.97 ± 3.41 and 5.44 ± 0.52 mmol/L (*p* < *0.001)*. Similarly, the HbA1c levels of the diabetic (7.61 ± 1.18%) and the obese (5.53 ± 0.29%) cohort differed significantly *(p* < *0.001)* as well. Additionally, we noted considerably higher levels of C- peptide and more pronounced hyperinsulinemia in the obese relative to the T2DM subjects (seen in Table 2; *p* < *0.05*).

### Perfusion studies

To explore potential differences between the peripheral microcirculation of T2DM and obese individuals, group comparison was performed. Lower limb SPECT studies revealed meaningful difference between the peripheral perfusion of the two examined groups *(SUVmean left leg: 0.53* ± *0.11 and 0.64* ± *0.13, for the diabetics and the obese; respectively and SUVmean right leg: 0.52* ± *0.09 and 0.6* ± *0.13 for the diabetics and the obese; respectively*), being higher on both sides in case of the obese individuals than in the diabetics (*p* < *0.001 and p* < *0.005 for left and right foot; respectively*).

In addition, correlation analyses were carried out for the assessment of the association between leg perfusion and BMI as well as laboratory biomarkers. Lower limb perfusion showed positive correlation with BMI *(p* < *0.001, rho* = *0.44)*, C-peptide *(p* < *0.05, rho* = *0.29)*, as well as insulin levels *(p* < *0.01, rho* = *0.30)*. On the contrary, HbA1c levels negatively correlated with the radiopharmaceutical uptake of the lower extremities (*p* < *0.05, rho* =—*0.24*). Although plasma glucose concentrations also showed inverse association with the leg SUV values, this was statistically not significant *(p* = *0.081, rho* =—*0.21).*

Regarding brain perfusion SPECT, neither the global nor the hemispheral cerebral perfusion of the two groups differed significantly *(p* > *0.05)*. Except for BMI *(p* < *0.005, rho* = *0.36)* and the C-peptide levels *(p* < *0.05, rho* = *0.27),* none of the assessed parameters were in significant correlation with central perfusion.

### Nerve conducting tests

Similarly to the perfusion examinations, group comparison and correlation analyses were conducted in case of the neurometer studies as well.

**Fig. 2:**
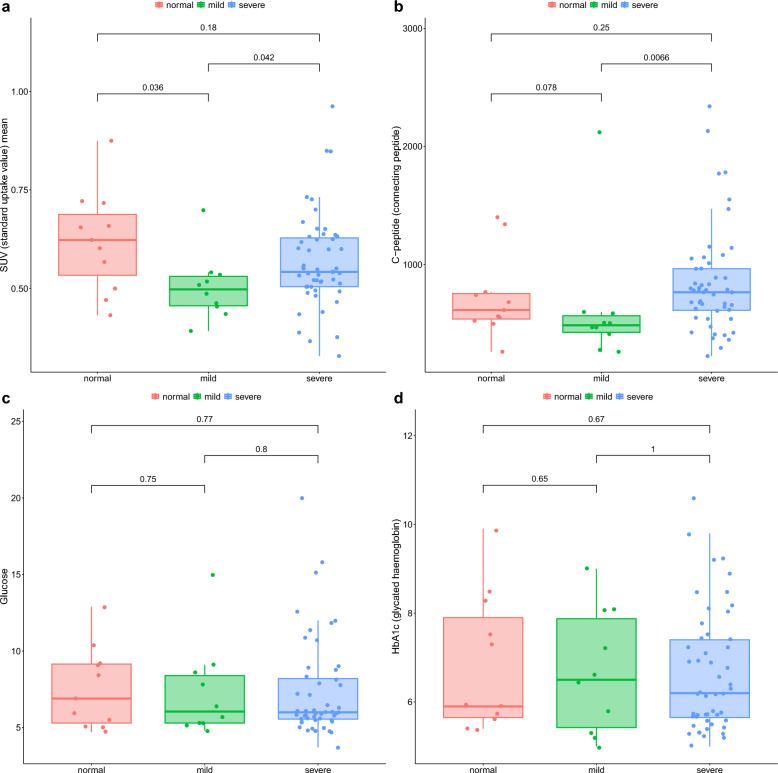
Box-and-whisker plots for the comparison of the lower limb perfusion (**a**), C-peptide (**b**), fasting glucose (**c**) and HbA1c (**d**) levels of the different neuropathy subgroups. Red points are for participants without neuropathy, green for those with mild neuropathy, and the blue ones indicate patients with severe neuropathy. The average radiotracer uptake of the lower extremities was expressed in SUV units. Statistical significance was established at *p* < *0.05*

Regardless of the type of metabolic disease present, the neurometer studies disclosed significant differences between the peripheral microcirculation of the different NP subgroups (Fig. 2, part a). First, the leg perfusion SPECT results of the mild-NP subgroup notably differed from those of the subcategory with no-NP, i.e. lower SUV values were measured for patients with mild-NP relative to those without nerve sensation loss. Intriguingly, we found considerably different SUV data/values between the mild and the severe-NP subgroups (*p* < *0.05*), being higher in the severe subgroup than in the mild-NP cohort. Although it did not seem to be statistically significant, the SUV values of the subgroup with severe-NP were lower compared to the subclass without NP *(p* = *0.18)*.

As Fig. 2, part b presents the change in C-peptide levels showed similar tendency to that of the lower limb microcirculation. Of note, participants with mild-NP were characterized by significantly lower C-peptide levels in comparison with the subjects with severe-NP (*p* < *0.01*). Additionally, compared to the subgroup without NP, lower C-peptide levels were observed in case of the patients with mild-NP, while higher values were measured in the severe-NP subgroup (*p* = *0.078, p* = *0.25; respectively*), however, these were statistically not significant.

**Fig. 3:**
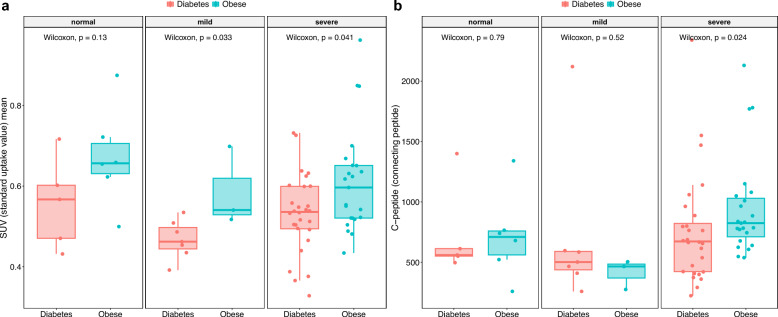
Box-and-whisker plots illustrate the lower limb perfusion and the C-peptide levels among diabetic and obese patients within different neuropathy subclasses in part **a** and **b**, respectively. Red points: diabetics, blue points: obese. Radiopharmaceutical uptake of the lower extremities was expressed in SUV units. Statistical significance was set at *p* < *0.05*

After categorizing the patients of the different NP subgroups based on the type of metabolic disease they had, significant distinctions were observed between the diabetics and the obese both in the mild and the severe-NP subgroups *(p* < *0.05)*. As indicated by Fig. 3, part a, patients with obesity in mild and severe-NP subgroups demonstrated notably improved lower limb microcirculation than the diabetic counterparts (*p* < *0.05*). Furthermore, significantly higher C-peptide levels were measured in the obese patients of the severe-NP subgroup compared to diabetics of the same subclass (*p* < *0.05,* Fig. 3, part b). Additionally, both the fasting blood glucose and the HbA1c levels showed significant differences when comparing the three NP subgroups (*p* < *0.001*, Table 1). Figure 2, part c and d, show no remarkable difference between the glucose and the HbA1c levels of the different NP subgroups (*p* > *0.05*). Additionally, the changes in these glycaemic indicators did not follow the same trend as those observed regarding lower limb microcirculation. Based on SPECT imaging and nerve conducting tests, no meaningful difference was noted between the brain perfusion of the three NP subcategories *(p* > *0.05)*.

Finally, upon correlation analyses with the baseline parameters, we did not observe any age-related differences between the subgroups with different degree of NP (*p* > *0.05,* as seen in Table 1). As for BMI, however, clear/evident distinctions were experienced between the obese and the diabetics regardless of the severity of NP *(p* < *0.001,* Table 1).

## Discussion

Although substantial progress has been made toward the understanding of how metabolic diseases affect nerve function and the microvasculature, several aspects remain still poorly understood. As better exploration of underlying pathophysiology may aid to combat disease-related health ramifications, in this multi-modal study we aimed to gain deeper insight into the relationship between the microcirculation, gylcaemic status and the peripheral nerve functioning of patients with T2DM and obesity.

Briefly, we observed that the severity of PN is strongly associated with the development of peripheral microvascular impairments in patients with T2DM and obesity. We also concluded that elevated C-peptide levels significantly affect both central and peripheral microcirculation in such patient cohorts. Lastly, strong association was experienced between C-peptide levels and peripheral microcirculation, that seemed to be dependent upon the severity of neuropathy.

### Perfusion studies

Significantly reduced lower limb perfusion in the diabetics compared to the obese subjects may indicate the presence of T2DM-related microvascular impairments in this patient cohort. Nevertheless, the exact reason why the obese participants presented better peripheral circulation relative to the diabetic group remains to be fully elucidated, we suppose that being a prediabetic condition, pathological microvascular changes induced by metabolic dysfunction are present to a lesser degree in obesity than in diabetes, that may provide some explanation. Our findings overall capitalize on the relevance of [99mTc]Tc-HMPAO SPECT/CT as a valuable tool to predict the development and the severity of peripheral perfusion changes associated with metabolic diseases. Although literature data report on the investigation of microcirculation in diabetes [27, 38, 39], only a few prior study applied nuclear medical techniques for such purposes [27, 40, 41] focusing mainly on patients diagnosed with PAD [40, 41]. According to prior findings contrast-free magnetic resonance imaging (MRI) [38], measurements of ankle-brachial index or transcutaneous tissue partial oxygen pressure [39] seem to be feasible for the assessment of disease-associated vascular alterations. Using MRI, Zheng et al. also identified diminished blood flow in diabetes, however, unlike our study, they involved a group of healthy people for comparison [38]. Non-invasive angiological and microrheological tests [39] revealed worse perfusion parameters in diabetic individuals with more severe disease stages compared to those presenting milder symptoms [39], that shows good correlation with the current observations. Although the results of these studies overlap with that of ours, given the differences between the patient populations and the applied methods we are reluctant to make direct comparisons. Digital photoplethysmography-based contrasting results indicating improved peripheral blood flow in T2DM, however, suggest the critical importance of the optimization of methodology to draw definitive conclusions [42].

In addition, glycaemic parameters and SPECT data were correlated to further evaluate the relationship between metabolic disturbances and lower limb microcirculation. As far as we are aware of the literature, our study is the first to discuss direct association of C-peptide levels with peripheral perfusion parameters. Although additional studies are required to confirm, we suggest that this may indicate the ability of this peptide to ameliorate vasculopathy induced by metabolic dysfunction. Considering former findings, C-peptide possibly exerts beneficial effects on the microvasculature via endothelial NO stimulation and the enhancement of red blood cell flexibility [36, 43], but its anti-inflammatory and anti-apoptotic role could not be ignored either [44]. Despite the significant positive correlation between insulin levels and lower limb perfusion, given that patients under regular insulin therapy were not excluded from the present study, further clinical implications could not be established. In line with earlier results HbA1c [27, 38], and serum glucose [27] levels negatively correlated with the perfusion of the lower extremities that could possibly be attributed to disrupted vascular signalling caused by hyperglycaemia and systemic met inflammation. Additionally, taking the associations between laboratory biomarkers and perfusion, we hypothesize that the glycaemic status of patients with metabolic diseases might anticipate the likelihood of the development of perfusion abnormalities.

Even though obese patients exhibited better leg perfusion compared to diabetics, no significant difference was found regarding their cerebral perfusion, and we hypothesize that this reflects the more pronounced vulnerability of peripheral microcirculation to disease-related hyperglycaemic effects that eventually leads to its earlier deterioration. Lack of negative correlation between glycaemic parameters and brain perfusion—unlike peripheral microcirculation—further confirms this suggestion. Intriguingly, Képes and colleagues [26] found perfusion differences between the same patient cohorts specifically in the region of the insula, that contradicts our findings. The fact that they focused on regional perfusion assessment while we currently analysed the global perfusion pattern could explain the distinctive observations. Similarly to their experiences [26], however, improved brain perfusion was observed with increasing BMI in our study as well that could presumably be attributable to central regulatory mechanisms. Moreover, in agreement with reports on the periphery, positive correlation of C-peptide levels and cerebral perfusion strengthens its unique contribution to the preservation of microvascular functions.

In view of these findings, we suppose that the integration of regular C-peptide measurements into clinical settings holds potential for the timely diagnostic assessment of microvascular damage in metabolic diseases along with personalized follow-up. This would not only allow for the selection of those patient population that might benefit from early therapeutic intervention but may revolutionize currently existing guidelines.

### Nerve conducting tests

To best of our knowledge, our study is the first to analyse peripheral and central perfusion patterns in association with the severity of PN in metabolic diseases. As expected, the mild-NP group showed notably reduced peripheral perfusion compared to the no-NP cohort, however, we surprisingly registered better perfusion parameters with worsening signs of neuropathy (mild-NP vs. severe-NP). Since changes in C-peptide and SUV data show a similar U-shaped trend across the different NP subgroups (as seen in Fig. 2, part a and b), a positive correlation between C-peptide concentrations and the extent of peripheral perfusion could be supposed that may aid in the interpretation of the experienced peripheral perfusion patterns. Therefore, the markedly higher C-peptide values of patients with severe-NP in comparison with the other two subgroups could provide a reasonable explanation for the better perfusion observed in the severe-NP cohort. Identically, lower C-peptide levels measured in the no-NP group compared to those with severe disease conditions could be the reason why the former (no-NP) group did not show more improved peripheral microcirculation than the severe-NP group. Additionally, in the obese cohort we suppose disease-related metabolic factors as well behind better perfusion in patients with severe NP. The above detailed collectively demonstrate—in line with literature findings [44–46]- that C-peptide exerts meaningful protective effect on the microvasculature in metabolic diseases. Furthermore, in obesity the axis of insulin-resistance, hyperinsulinaemia and subsequent elevated C-peptide levels must be addressed behind this phenomenon. Unlike C-peptide, other glycaemic indicators (glucose, HbA1c) did not follow a similar U-shaped pattern, and no significant difference could be depicted between these parameters of the three NP subgroups. Considering literature data that indicates that NP development might possibly be independent of glycaemic control [47], our result may highlight the potential superiority of C-peptide in vascular regulation over blood glucose and related glycaemic markers. It is worth emphasizing, however, that the present study population was under regular medical control to maintain glucose homeostasis within a desirable/acceptable range that presumably influences the results of the correlation tests. Nevertheless, C-peptide plays a major role in the fait of NP, given that the pathophysiology of NP seems to differ between obesity and T2DM [48, 49], biomarkers beyond C-peptide are also required for more precise assessment. In DM vascular factors are the major contributors to NP progression, while as for obesity metabolic aspects appear to be the dominant [50, 51], therefore molecular investigations into these specific fields may herald a new era in disease diagnostics and prevention.

Although there is a shortage in prior publications on microcirculation in neuropathy, similar findings of Tomesová et al. indicating impaired microcircular reactivity in diabetic neuropathy are worth mentioning [52]. Conversely, no associations were experienced between neuropathy indexes (CPT) and lower limb perfusion in the study of Képes et al. with T2DM and obese people, although their analyses were independent of the severity of NP [27]. Further in their research, HbA1c levels showed strong positive correlation with nerve conduction in diabetes, that also contrasts with the present findings [27].

Similar results on SPECT perfusion tests—indicating better performance and higher C-peptide levels for the obese than the diabetics in all NP subcategories—to those of the group comparison further support the promising biological activity of C-peptide on microvascular function. Former proof-of-concept study had matching results [27] when comparing the peripheral perfusion of non-DM obese individuals to that of people with T2DM.

Finally, lack of difference between the brain perfusion of the no-, mild-, and severe-NP groups let us draw the conclusion that—unlike peripheral microcirculation—central perfusion is independent of PN. Based on this, we also speculate that alterations in the peripheral vasculature presumably precede cerebral changes in NP suggesting the better adaptability of the brain.

## Conclusion

The present study provides a comprehensive analysis on the effects of T2DM and obesity on the microvasculature and peripheral nerve functioning. Indicating significant association of peripheral and central perfusion and PN via C-peptide, we have provided a strong and scientifically justifiable rationale for the application of this peptide hormone in routine diagnostic work-up. Although the exploration of the complex pattern of the link between C-peptide and microvascular changes is part of future work, our findings highlight its protective effects against microcircular complications related to metabolic disturbances. This observation will pursue to broaden the horizons of research in such key areas as endocrinology, contributing not only to better capture the totality of disease pathophysiology, but also ensuring unique opportunity for the discovery and evaluation of candidate drugs.

## Data Availability

No datasets were generated or analysed during the current study.

## Abbreviations

[^99m^Tc]: Technetium-99 m
BMI: Body Mass Index
BW: Body weight
cm: Centimeter
C-peptide: Connecting peptide
CPT: Current perception threshold
CT: Computed tomography
HbA1c: Glycated haemoglobin
HMPAO: Hexamethylpropylene amine oxime
HPLC: High-performance liquid chromatography
kg: Kilogram
m^2^: Square meter
mild-NP: Mild neuropathy
NaF–KOx: Sodium fluoride-potassium oxalate
NO: Nitric oxide
no-NP: No neuropathy
NP: Neuropathy
PAD: Peripheral arterial disease
PN: Peripheral neuropathy
SD: Standard deviation
severe-NP: Severe neuropathy
SPECT: Single-photon emission computed tomography
SPECT/CT: Single-photon emission computed tomography/computed tomography
SUV_mean_: Mean standardized uptake values
T2DM: Type 2 diabetes mellitus
VOIs: Volumes of interest
